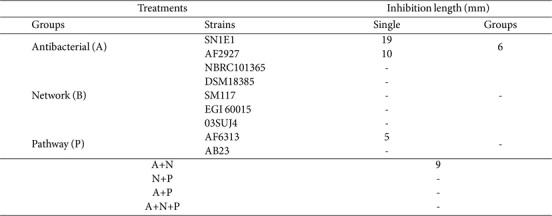# Design and Evaluation of Synthetic Microbial Communities for Effective Fire Blight Disease Control in Apples

**DOI:** 10.4014/jmb.2501.01047

**Published:** 2025-04-11

**Authors:** Yejin Lee, Da-Ran Kim, Youn-Sig Kwak

**Affiliations:** 1 Division of Applied Life Science (BK21), Gyeongsang National University, Jinju 52828, Republic of Korea; 2 National Institute of Horticultural and Herbal Science, Rural Development Administration, Wanju 55365, Republic of Korea; 3 Institure of Agriculture and Life Science, Gyeongsang National University, Jinju 58282, Republic of Korea

**Keywords:** Antibacterial, *Erwinia amylovora*, keystone taxa, microbial metabolic pathway, SynCom

## Abstract

Fire blight disease caused by *Erwinia amylovora* has resulted in extreme economic losses to industrial plants of the Rosaceae family, including apples and pears, since the 1870s. Many countries have used pesticides and bactericides to manage the fire blight disease. However, chemical management leads to the emergence of a pesticide-resistant pathogen population. Therefore, attempts at managing the fire blight disease have been developed and applied using selected microorganisms as a biological control. However, a single strain of the biological control agent showed limited effect in reducing the disease. Here, we designed synthetic microbial communities (SynCom), which involve reconstituting multi-strains rather than relying on a single strain to enhance the disease control efficiency. We constructed the SynCom based on three categorized functions: i) anti-EA strain, ii) keystone taxa in healthy apples, iii) abundant metabolite strain in healthy apples than the fire blight infected apples. SynCom was significantly efficient in suppressing fire blight, achieving 0% disease severity in apple fruits, 1.67% in roses, and 5.4% in apple plants. Our finding presented that the well-designed SynCom showed significant effects in controlling the fire blight disease. Additionally, SynCom members should be selected based on multiple functions to maximize the impact on crop management.

## Introduction

Apple (*Malus pumila*) is a globally cultivated a fruit known for their high content of flavonols, anthocyanins, dihydrochalcones, quercetin, catechins, tannins, and dietary fiber [1, 2]. However, apple cultivation faces a formidable challenge from fire blight disease, which significantly diminishes both yield and fruit quality [3, 4]. Once infected with this disease, apple trees pose a risk of orchard-wide devastation, complicating overall operational management. In high-density orchards, the infection rate is elevated, leading to an average loss of 2,500 trees per orchard [5]. According to the 2023 EPPO database, fire blight disease has been spread to over 50 countries across Europe, North Africa, North America, Asia, and Oceania. *Erwinia amylovora* is a gram-negative bacterial pathogen responsible for causing the fire blight disease. The symptom shows dark, scorched lesions, bacterial ooze, and cankers on species within the Rosaceae family such as apple and pear (*Pyrus pyrifolia*) [6]. *E. amylovora* has a type III secretion system (T3SS), which is a needle-like structure that plays a crucial role in transferring important virulence-effector proteins to the host cells [7, 8]. The infection primarily begins at the flower stigma, where the bacteria form clusters of exopolysaccharides (EPS), leading to both internal and external spread within the plant [9, 10]. The bacterial ooze acts as a transmission vector through pollinating insects and inanimate surfaces, enabling continuous contagion [10, 11].

*E. amylovora* has historically been controlled primarily using antibiotics, such as streptomycin sulfate. However, the emergence of antibiotic-resistant strains necessitated the exploration of alternative management technology [12, 13]. Moreover, within the societal trend emphasizing environmental conservation, reducing the use of antibiotics such as streptomycin is imperative [14]. In alignment with this trend, biological control utilizing microbial agents has been extensively studied to control fire blight disease [15]. Particularly, elucidating the potential pathogen inhibition and immune response mechanisms inherent in plant endophytic microorganisms has facilitated the use of biological control, leveraging the diverse bioactivities of endophytes [16].

Synthetic communities (SynCom) refer to the cultivation of multiple taxa together to mimic the desired structure and function of microbiota communities [17]. In contrast to conventional approaches that rely on individual microbes with either antibacterial properties or plant growth-promoting effects, SynComs aims to enhance its functions by simultaneously harnessing multiple microbial members [18, 19]. This study aimed to construct and utilize SynComs based on taxa selected in apple. To mimic complex microbial communities, SynComs were constructed by an analysis of microbial interactions and antibacterial activity tests against the pathogen.

In our previous work, we secured strains with anti-*E. amylovora* activity and analyzed microbial structure in healthy and *E. amylovora*-infected apples [20, 21, 22] Through correlation network analysis based on microbial density and metabolic pathway analysis, we gained insights into competitive relationships among microbes for nutrients metabolized by *E. amylovora* [22]. These competitive relationships concerning nutrients and spatial factors contribute to reducing microbial density [23]. Also, we identified the taxa associated with distinct metabolic pathways contingent on the presence or absence of fire blight infection [20]. Building on these findings, the study proposes a framework for designing SynCom with potential fire blight disease-suppressing effects.

## Material and Methods

### Antibiotic-Related Enzyme Activity of SynCom Strains

In this study, a total of nine individual strains were employed to evaluate the suppression of fire blight. Two of the nine were selected for their antibacterial activity, five were nominated keystone taxa based on the apple microbiome network, and two were obtained as microbiota community metabolite-responsible agents. Table 1 presents the SynCom members and references. We conducted a Chrome Azurol S (CAS) assay using a siderophore selection medium to evaluate siderophore production capacity and facilitate trace element iron absorption. The medium, as described by Louden *et al*. [24], was composed of 100 ml MM9 salt solution (750 ml ddH_2_O, 32.24 g PIPES, and 15 g agar/L, pH 6.5. After sterilization, 30 ml casamino acid solution was supplemented at 50°C). Additionally, 10 ml of 20% glucose solution and 100 ml blue dye solution (0.6 g chrome azurol S, 0.027 g FeCl_3_∙6H_2_O and 0.73 g Hexadecyltrimethylammonium bromide/L) were incorporated. Inoculating 20 μl of OD_600_ 0.5 SynCom strains, we measured clear zones after the 7-day incubation period.

To assess protease production, skim milk agar media (10 g skim milk powder, 20 g agar/L) were employed as the substrate [25]. Each strain (20 μl of OD_600_ 0.5) inoculum was applied to the media. Following incubation at 28°C for 3 days, the evaluation of proteinase activity was conducted by observing the development of a clear zone resulting from the degradation of proteins within the skim milk media. To test cellulase production ability, 1%carboxymethyl cellulose (CMC) medium with the following composition (1 g yeast extract, 1 g CMC, 1 g KH_2_PO_4_, 1g MgSO_4_, 0.05 g MnSO_4_, 0.05 g FeSO_4_, 2 g CaCl_2_, 2 g NH_4_Cl and 20 g agar/L) was adjusted to a pH of 7.0~7.4 [26]. Each strain of 20 μl with an OD_600_ 0.5 was inoculated on a paper disk in three directions on the plate. After incubation at 28°C for 48 h, approximately 5 ml of Gram’s iodine solution (0.67% KI, 0.33% I_2_) was poured onto the plate and allowed to stain for 5 min. To evaluate chitinase activity, chitinolytic reaction media (5 g chitin, 5 g yeast, 0.7 g KH_2_PO_4_, 0.05 g K_2_HPO_4_, 0.3 g MgSO_4_, 0.1 g FeSO_4_, 0.1 g NaCl and 20 g agar/L, pH 6.5~7.0) was prepared at the same concentration as the cellulase experiment and cultured for 3 days [27]. Then, 1 ml of 0.1% Congo Red was dispensed per plate and incubated for 15 to 30 min, followed by drying for a while. Subsequently, the washing process was repeated twice by dispensing 2 ml of 1 M NaCl, and the clear zone was measured.

To measure nitrogen fixation ability, nitrogen-free bromothymol blue broth (NFb; 5 g malic acid, 0.6 g K_2_HPO_4_, 0.4 g KH_2_PO_4_, 0.02 g NaCl, 0.002 g Na_2_MoO_4_∙2H_2_O, 0.01 g MnSO_4_∙H_2_O, 2 ml bromothymol blue 0.5% in ethanol, 1.75 g agar/L, pH 6.6~7.0) 5 ml of media was inoculated and cultured at 28°C for 72 h [28]. Since the medium lacks a nitrogen source, only strains that can fix atmospheric nitrogen can grow, so the nitrogen fixation ability can be evaluated according to the OD_600_ value.

### Plant Growth Promotion-Related Activity of SynCom Strains

The ability to form biofilms, which is crucial for establishing colonization within plant tissues, was assessed [29]. Strains were cultured in R2A media (0.5 g casamino acid hydrolysate, 0.5 g yeast extract, 0.5 g proteose peptone, 0.5 g dextrose, 0.5 g soluble starch, 0.3 g dipotassium phosphate, 0.05 g magnesium sulfate, 0.3 g sodium pyruvate and 15 g agar/L, pH 7.0 ~7.4) with 5% mannitol at 28°C with shaking for 2 days. The cultures were then diluted 1/10 in M63 media (2.0 g (NH_4_)_2_SO_4_, 13.6 g KH_2_PO_4_, 0.5 mg FeSO_4_·7H_2_O, after autoclave, 10 ml sterile 20% solution of glycerol and 1 ml sterile 1M MgSO_4_ solution/L, pH 7.0) and dispensed 100 μl per well into a 96-well plate. After incubating without shaking for 24 h, wells were washed twice with sterile water and stained with 125 μl of 0.1% crystal violet for 10 min at room temperature. Wells were then washed 4 times with sterile water and dry for overnight. Finally, 200 μl of 95% ethanol was added to each well, and after 10 min, the OD_600_ was measured.

To ascertain the biosynthesis of indole-3 acetic acid (IAA), the Salkowski method was employed [30]. SynCom strains were cultured in R2A+0.5% mannitol media at 30°C for 3 days, either with or without 3 mM tryptophan. SynCom strains were cultured in R2A media supplemented with 0.5% mannitol at 30°C for 3 days, with two treatment groups: one with the addition of 0.1% L-tryptophan and the other without L-tryptophan. Subsequently, the cultures were centrifuged at 3,393 ×*g* for 30 min to separate the supernatant. The supernatant was then mixed with Salkowski reagent (2% of 0.5 M FeCl_3_ in 35% HClO_4_ solution) in a 1:2 ratio and incubated at room temperature for 30 min. A color change from orange to pink indicates the production of IAA. OD was measured at a wavelength of 530 nm.

To measure phosphate solubilization ability, bacteria were screened using Pikovskaya medium (0.2 g KCl, 0.1 g MgSO_4_·7H_2_O, 10 g glucose, 0.5 g yeast extract, 0.002 g FeSO_4_·7H_2_O, 0.002 g MnSO_4_, 0.5 g [NH_4_]_2_SO_4_ and 5 g Ca_3_[PO_4_]_2_/L) [31]. Bacteria were inoculated onto paper disks with 20 μl adjusted to OD_600_ of 0.5. The plates were then monitored for approximately 2 weeks at 28°C to observe the formation of clear zones around the disks.

### *In Vitro* Evaluation of Anti-*E. amylovora* Activity of SynCom

In this assay, a confrontation culture was conducted to assess the inhibitory effects of SynCom against *E. amylovora* TS3128 [32]. The treatment was divided into a single strain and the group (A: antibacterial, N: network, P: pathway, A+N, N+P, A+P, and A+N+P) categories. All strains were uniformed to an OD_600_ of 0.7. Subsequently, 20 μl of each strain was dotted onto paper disks placed on R2A agar media supplemented with 0.5%mannitol. The plates were incubated for 3 days. For anti-*E. amylovora* assessment, strains belonging to each group were mixed in equal concentrations and volumes to create group-specific mixtures. In cases of combined groups, mixtures from separate groups were combined in equal amounts, dotted 20 μl onto media, and incubated for 3 days. *E. amylovora* was cultured in MGY broth (10 g D-mannitol, 2 g L-glutamic acid, 0.5 g KH_2_PO_4_, 0.2 g NaCl, 0.2 g MgSO_4_∙7H_2_O, 1 g yeast extract and 15 g agar/L, pH 7.0) for one day, washed twice with sterile water, and adjusted to an OD_600_ of 0.2. Sterilized agarose 0.2% was mixed with the culture in a 1:1 ratio, dispensed onto plates (2 ml per plate), dried, and observed for clear zones 2 days later.

### Fire Blight Disease Suppression Assay by the SynCom in Apple Fruits

*In planta* biological control assay for fire blight disease suppression in apple fruits involved nine experimental groups: untreated, EA (*E. Amylovora*), EA+each single group (A, N, P) inoculation, EA+combinations of two groups (A+N, N+P, A+P), and EA+three-groups combination (A+N+P). All strains were suspended in sterile water and subsequently diluted to an OD_600_ of 0.2. For each group, an equal amount of each strain was combined, and then 1% CMC was added in a 1/10 ratio.

Apple fruits (cv. Fuji) were sterilized in 1% NaOCl and then washed twice with sterilized water and cut in half. The central part of the round side of the cut apple was perforated with a 4 mm cork borer, and the hole was inoculated with the SynCom for each group and *E. amylovora*. 50 μl of the prepared SynCom mixture was inoculated and dried for one day. *E. amylovora* was cultured in MGY broth for approximately 24 h before inoculation. One day later, when the mixture dried, 20 μL of *E. amylovora*, washed and diluted with 10% sucrose, with an OD_600_ of 0.1, was dispensed. Disease severity was assessed based on symptoms of discoloration and necrosis. Around 10 days post-treatment with the pathogen, the assessment of disease severity utilized a scale from 0 to 5. Disease severity index (DSI) represents different levels of symptom in the study as follows: Index of 0 indicated no symptom, 1 indicated water-soaked lesions covering 1-5% with browning ranging from 1-3%. Index of 2 indicated water-soaked lesions covered 1-5% with browning extending to 4-10%, 3 indicated water-soaked lesions ranging from 15-50% with browning spanning 11-30%, 4 indicated 51-70% coverage of water-soaked lesions and 31-60% browning. Lastly, 5 was designated for cases with 71-100% coverage of water-soaked lesions and 61-100% browning. DSI (%) = [sum (class frequency × score of rating class)] / [(total number of plants) × (maximal disease index)] × 100. These formulas were employed to assess DSI in the study. DSI represents the overall severity of symptoms, calculated by summing the product of class frequency and the score of the rating class, divided by the product of the total number of plants and the maximal disease index. The result was multiplied by 100 to express it as a percentage. These standardized metrics provide a comprehensive evaluation of disease severity and incidence in the experimental setting. Each group was treated with 15 replicates. Statistical analysis was performed using the Kruskal-Wallis Rank Sum Test and Benjamini-Hochberg (BH) post-hoc analysis.

### Fire Blight Disease Preventing by the SynCom in Rose Flowers

The disease suppression in rose flowers aimed to observe the overall control efficiency of the SynCom. Treatment groups were identical to those in the apple fruit assay, with an additional positive control group EA+oxytetracycline (0.25 g/l, a. i. Oxytetracycline dihydrate 34%). For flowers, a 5 ml falcon tube containing 4 ml of 10% sucrose was inserted into wet floral foam soaked in sterile water, and then the stem was placed in the tube. SynCom mixture was prepared following the same procedures as in previous assay using fruits. Approximately 50 μl of SynCom and oxytetracycline (0.5 g/l) were inoculated into the flower ovary with syringe, and wet floral foam was placed in a cage containing sterilized water to provide moisture with sterilized water. For *E. amylovora*, 2.5 M MgCl_2_ was added in an amount of 1/10 to the diluted solution adjusted to OD_600_ of 0.5, and 20 μl of each flower was inoculated under the ovary on the opposite side treated with the SynCom mixture. After 9 days, DSI was measured from 0 to 5. The Disease Severity Index represents different levels of symptom in the study as follows: 0 indicated the absence of symptoms, 1 indicated light browning of the ovary, 2 indicated browning of 100% of the ovary and less than 30% of the calyx, and 3 indicated 100% of the ovary and less than 30% of the calyx. Browning of more than 30% of the calyx was set as the standard, 4 indicated browning of 100% of the ovary and less than 50% of the calyx, and 5 indicated browning of more than 100% of the ovary and 50% of the calyx. The assay was conducted with 12 repetitions per each group. The significance test was Kruskal-Wallis Rank Sum Test and BH post-hoc analysis.

### Evaluation of Suppressiveness Fire Blight Disease by the SynCom in Apple Plants

The assays utilizing apple plants were conducted two completely independent experiments, employing distinct methods of SynCom and pathogen inoculation. The treatment groups were the same as the fruits and flowers assays. The first assay was conducted with 15 repetitions for each group. The mixed SynCom was evenly sprayed onto the shoots of apple at a rate of 50 ml per treated group. The leaves and stems were gently rubbed to allow SynCom to penetrate the plants through stomata or wounds. For *E. amylovora* inoculation, after 24 h, the tip of the actively growing terminal bud was inoculated by cutting the leaf just below the first undeveloped leaf using scissors pre-soaked in a suspension of the *E. amylovora* strain diluted in 10% sucrose to OD_600_ of 0.2. After 7 days, disease symptoms on apple plants were assessed. An index ranging from 0 to 5 was employed to evaluate disease severity. The DSI represents different levels of symptom manifestation in the study as follows: 0 indicated the absence of symptoms, 1 indicated partial necrosis of the shoot tip, 2 indicated complete necrosis of the shoot tip, 3 indicated partial necrosis of the terminal leaves, 4 indicated complete necrosis on the petiole of terminal leaves, and 5 indicated complete necrosis on the main stem. Statistical analysis was conducted using the Kruskal-Wallis test followed by BH post-hoc analysis.

The second assay was conducted through 30 repetitions, where treatments were prepared in the same manner, with the inoculation method being altered. Incisions were made at the branching points of the apple stem, and the designated SynCom mixture was applied using a sterilized brush. After 3 h, once the SynCom had dried, the pathogens were inoculated onto the apple seedlings in the same manner as with the SynCom. After 5 days, DSI and statistical analysis were evaluated based on the same DSI as in the first assay.

### Microbial Community Structure Analysis after Introducing the SynCom

We observed whether endogenous microbial community changes or not in SynCom treated apple plants to assess environmental stability. To investigate whether SynCom strains induce microbiota alterations within the plant, we extracted DNA from both stem and root endosphere of apples. For the analysis of endophytic microorganisms in stems and roots, external microorganisms were disinfected through a washing procedure. The samples underwent sequential washing steps with 1% NaOCl for 30 sec., followed by 70% ethanol for 30 sec., and rinsed twice with sterile water. Subsequently, the samples were air-dried. DNA extraction was performed using the FastDNA Spin Kit for Soil (MP Bio, USA) with five repetitions. For next generation sequencing (NGS) analysis, specific regions were amplified using KAPA HiFi HotStart ReadyMix (Roche, Swiss). Primers 515F (TCGTCGGCAG CGTCAGATGTGTATAAGAGACAGGTGYCAGCMGCCGCG-3’) and 805R (GTCTCGTGGGCTCGGAGAT GTGTATAAGAGACAGGACTACHVGGGTATCTAATCC-3’) containing adapter sequences recognized by the equipment were used. We conducted a blocking polymerase chain reaction (PCR) to enhance specificity for microbial 16S rRNA V4 region amplification. To prevent amplification of plant’s mitochondria and chloroplast regions, the blocking primers pPNA (5’ GGCTCAACCCTGGACAG-3’) and mPNA (5’ GGCAAGTGTTCTTCGGA-3’) were employed to selectively clamp these regions [33]. The components in the first-round PCR reaction mixture for amplifying the targeted region included 5-8 ng/μl DNA, 515F primer (10 pmol), 805R primer (10 pmol), pPNA (7.5 μM), mPNA (7.5 μM), ddH_2_O, and KAPA HiFi HotStart ReadyMix. The PCR conditions consisted of an initial denaturation at 98°C for 3 min., followed by 25 cycles of denaturation at 98°C for 30 sec., annealing at 55°C for 30 sec., extension at 72°C for 30 sec., and a final extension at 78°C for 10 sec. The obtained fastq files underwent analysis following the DADA2 version 1.8 guidelines (https://benjjneb.github.io/dada2/tutorial_1_8.html). Sequences with quality scores above 30 were trimmed, and forward and reverse reads were merged after removing chimeras. Each library was taxonomically classified into phylogenetic groups with 95% or higher similarity using Silva release 138 (https://www.arb-silva.de/) and IDTAXA. Alpha and beta diversity were visualized using R packages such as ggplot2 (version 3.4.2) and ggh4x (version 0.2.6). For the alpha diversity analysis, the Observed index, Chao1 index, and Shannon index were employed. Principal Coordinates Analysis (PCoA) was conducted using the Bray-Curtis dissimilarity method to examine variations in bacterial communities across SynCom treatment. Additionally, pairwise adonis (version 0.4.1) was utilized for permutational multivariate analysis of variance (PERMANOVA) to statistically assess whether SynCom group treatments induced significant differences in bacterial communities.

## Results

### SynCom Members Acquisition through Microbiome Analysis

Among the antibacterial group strains, *Paenibacillus polymyxa* AF2927 was isolated from apple flower endophytes [22], and *Streptomyces recifensis* SN1E1 was isolated from soil [34] (Table 1). For the network group, strains with the highest sequence similarity to the selected OTUs were obtained from the Korean Agricultural Culture collection (KACC). The genus *Labrys*, identified through network analysis, showed 97.0149% alignment in the 16S rRNA region with *Labrys miyagiensis* and *Labrys okinawensis* [22]. OTUs identified as *Novosphingobium* in the network analysis were aligned with *Novosphingobium mathurense* with 100% sequence similarity and *Novosphingobium endophyticum* with 98.7562% similarity. For the genus *Terriglobus*, the *T. aquaticus* 03SUJ4 strain showed 99.88% alignment with OTU. In the pathway group, *Kitasatospora papulosa* AF6313 was isolated from an apple flower endophyte [21], and *Pseudomonas lundensis* AB23 was isolated from bee gut in apple orchards. Both strains exhibited 100% alignment with the ASV contributing to the reference pathway [20].

### SynCom Has Diversity in Plant Growth-Promoting Activity

Microorganisms possessing enzyme activity have been reported to exhibit either plant growth-promoting effects or inhibit growth through the dissolution of plant pathogens. Therefore, in this study, we measured the activities of cellulase, siderophore, proteinase, chitinase, nitrogen fixation, biofilm formation, IAA production, and phosphate solubilization in SynCom strains (Figs. S1-S8). The results of the enzyme activity tests revealed that *S. recifensis* SN1E1 and *P. polymyxa* AF2927, both belonging to the antibacterial group with high antibacterial activity, and elevated cellulase activity (Figs. S1, S9). *K. papulosa* AF6313, while demonstrating less cellulase activity compared to antibacterial group strains, still displayed a notable pattern of cellulase activity (Fig. S1). However, the strain showed no activities for siderophore and chitinase production (Figs. S2, S3). Protease production ability was most pronounced in *K. papulosa* AF6313, followed by *P. polymyxa* AF2927 (Fig. S4). Nitrogen fixation and biofilm formation abilities were statistically significantly highest in *P. lundensis* AB23 and *L. miyagiensis* NBRC101365, respectively (Figs. S5, S6). In IAA production, *T. aquaticus* 03SUJ4 exhibited the highest activity, while *L. miyagiensis* NBRC101365, within the same network group, also showed statistically high differences in activity (Fig. S7). Additionally, *N. mathurense* SM117 and *N. endophyticum* EGI60015 from the network group showed lower activity compared to the *L. miyagiensis* NBRC101365. In the phosphate solubilization, *P. polymyxa* AF2927 from the antibacterial group exhibited a clear zone of 2 mm, indicating weak phosphate solubilization ability. In comparison, *K. papulosa* AF6313 from the pathway group also displayed weak phosphate solubilization ability with a clear zone less than 1 mm (Fig. S8).

### The Varying Anti-*E. amylovora* Effects of SynCom Member Strains

In the assessment of antibacterial effects against EA under *In vitro* conditions, the assays were conducted by inoculating individual strain and groups, followed by overlaying *E. amylovora* for the comparison of anti-EA activity, observed as clear zones (Table 2, Fig. S9). The untreated group served as the control, and EA represented the reference group treated only with *E. amylovora*. *S. recifensis* SN1E1 from the antibacterial group exhibited the highest anti-EA activity among all strains. *P. polymyxa* AF2927 from the antibacterial group, while less potent than SN1E1, still demonstrated a clear zone of approximately 10 mm. Strains from the network and pathway groups exhibited negligible anti-EA activity, while *K. papulosa* AF6313 displayed weak anti-EA activity, with a clear zone length of approximately 5 mm. In the SynCom groups, various combinations denoted as A (antibacterial), N (network), P (pathway), A+N, N+P, A+P, and A+N+P were used. The results of observing anti-EA effects by group showed that only groups A and A+N exhibited anti-EA activity, forming clear zones of 6 mm and 9 mm, respectively. In these two groups, the dominance of *P. polymyxa* AF2927 from the antibacterial group was confirmed to have grown.

### SynCom Inhibited the Fire Blight Disease in Apple Fruits

**Fig. 1:**
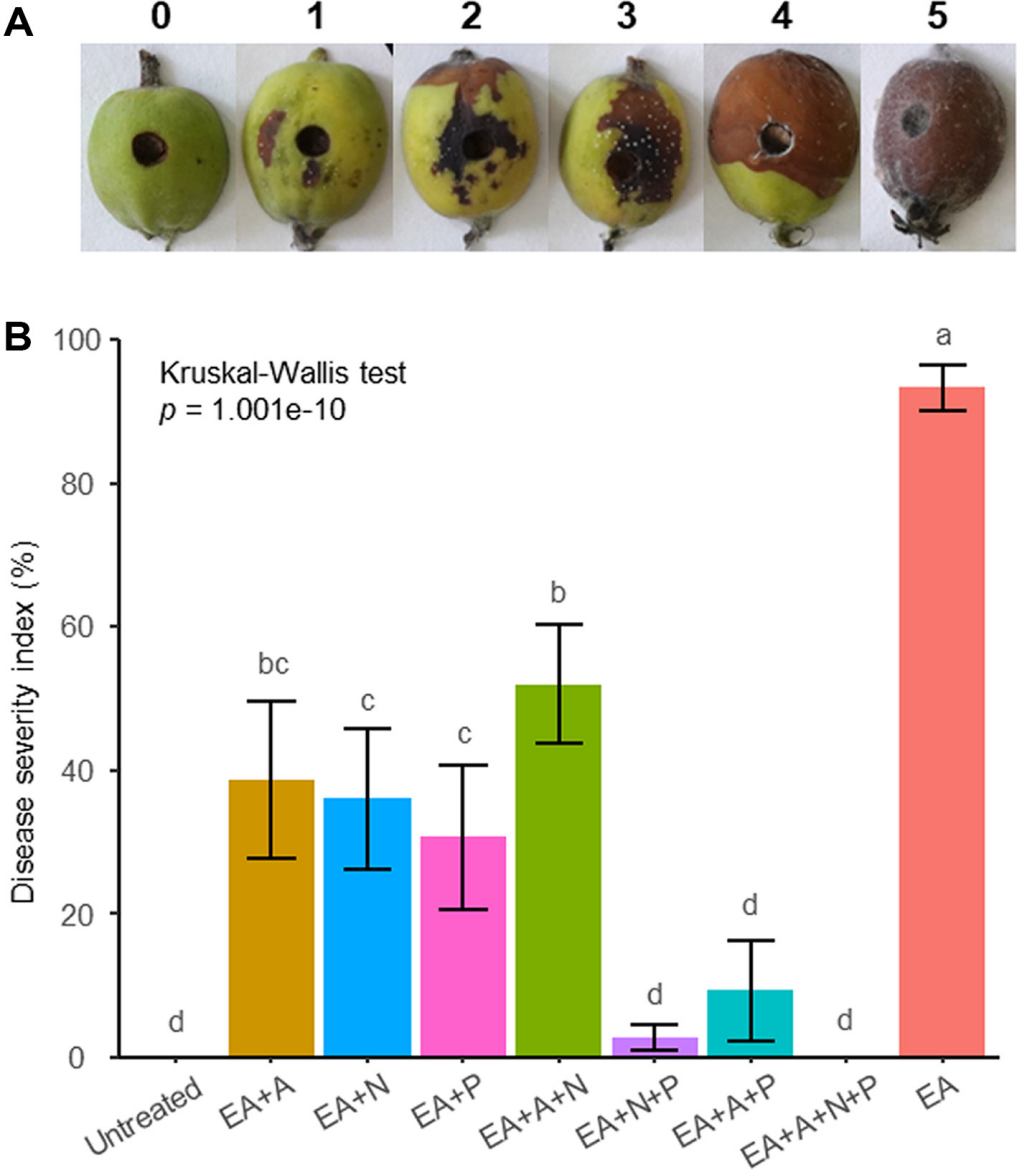
A biological control assay using apple fruits (*n* = 15). (**A**) Disease severity index of apple fruits. Index 0: No symptoms, Index 1: Water-soaked lesions covering 1-5% with browning ranging from 1-3%, Index 2: Water-soaked lesions covering 1-5%, with browning extending to 4-10%, Index 3: Water-soaked lesions ranging from 15-50% with browning spanning 11-30%, Index 4: 51-70% coverage of water-soaked lesions and 31-60% browning, Index 5: 71-100% coverage of water-soaked lesions and 61-100% browning. (**B**) Represent for assessment of fire blight severity on apple fruits. The apple fruits were punctured using a 4-mm cork borer. OD_600_ of 0.2 SynCom strains was mixed for each group, and then mixture was created by adding 1/10 volume of CMC. 50 μl of SynCom mixture was injected into each fruit. One day later, 20 μl OD_600_ of 0.1 *E. amylovora* was treated on the same area, and the severity of the disease was confirmed 10 days later. EA: *E. amylovora*, A: antibacterial, N: network, P: pathway. Kruskal-Wallis test was conducted to investigate differences among treated groups. In cases where a significant difference was detected at the 5% significance level, post hoc Conover-Iman tests with BH correction were performed for multiple pairwise comparisons.

In the experimental validation of the control efficacy against fire blight in young apple fruits, it was observed that discoloration, exudation of bacterial fluid, and water-soaked lesions were evident at the pathogen-inoculated sites (Fig. 1A). Disease severity was evaluated based on the areas of discoloration and water-soaked lesions. The results of the assay demonstrated that anti-EA activity was observed in all SynCom treated groups (Fig. 1B). In terms of severity, while the control group with only *E. amylovora* (EA) exhibited 93.33%, the SynCom treated groups showed varying degrees: EA+A (antibacterial)+N (network) at 52%, EA+A at 38.67%, EA+N at 36%, EA+P (pathway) at 30.67%, EA+A+P at 9.33%, EA+N+P at 2.67%, EA+A+N+P at 0% severity, and control at 0%, in descending order. The SynCom treatments exhibited a consistent and substantial control effect of at least 40%, and the treatment group where all groups were combined demonstrated a 0% incidence rate, indicating no infection by *E. amylovora*.

### Fire Blight Disease on Flower Was Controlled by the SynCom

**Fig. 2:**
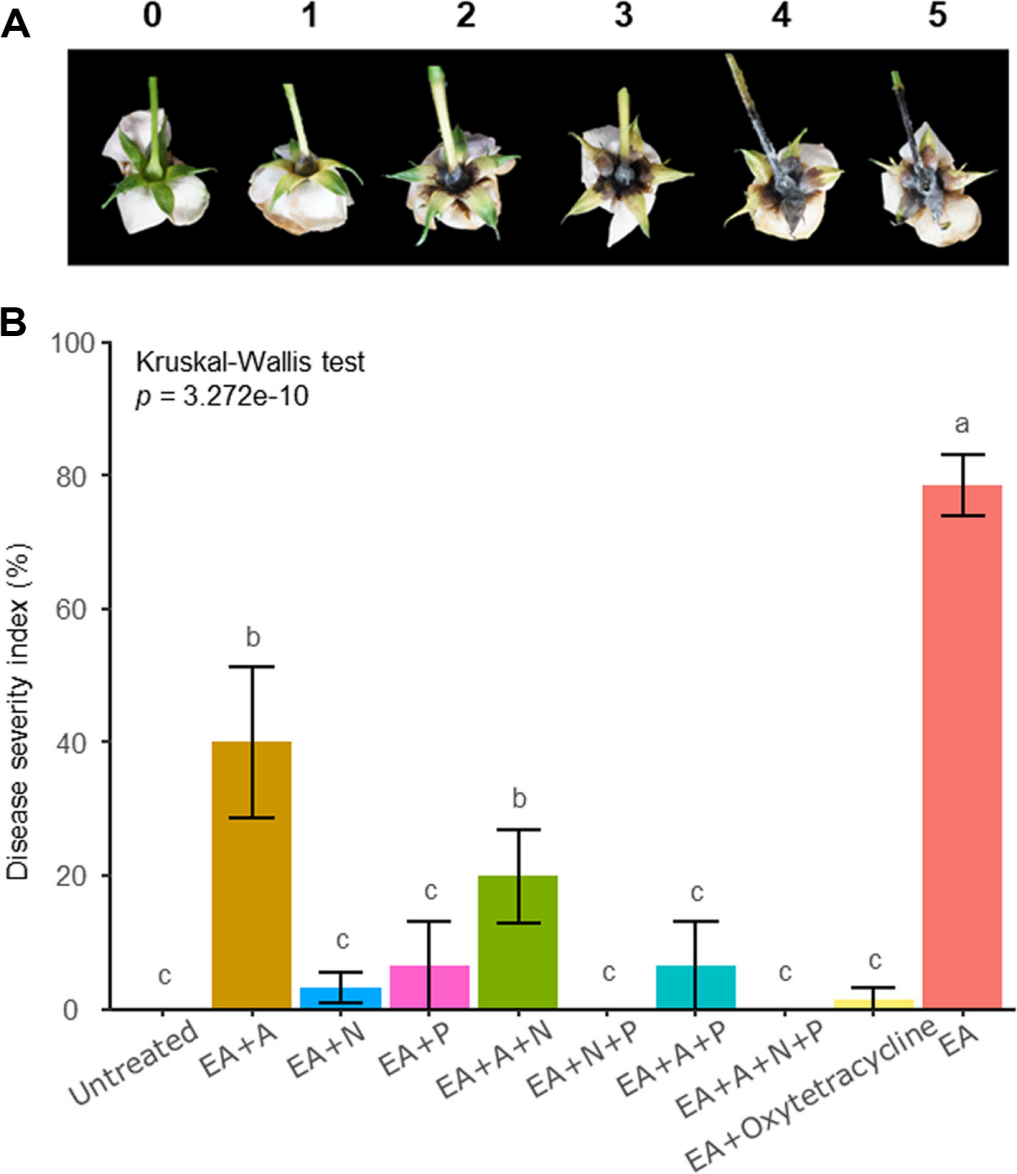
The impact of SynCom on fire blight disease suppression in roses (*n* = 12). (**A**) Disease severity index in rose flowers. Index 0: No symptoms, Index 1: Light browning of the ovary, Index 2: Browning of 100% of the ovary and less than 30% of the calyx, Index 3: 100% browning of the ovary and less than 30% of the calyx, with browning more than 30% of the calyx as the standard, Index 4: Browning of 100% of the ovary and less than 50% of the calyx, Index 5: Browning of more than 100% of the ovary and 50% of the calyx. (**B**) Represent for assessment of fire blight severity on roses. SynCom strains at OD_600_ of 0.2 were mixed in equal amounts and combined with 1/10 of 1% CMC. A 50 μl volume of this mixture was injected into flower ovaries. *E. amylovora* was prepared at OD_600_ of 0.5, mixed with 1/10 of 2.5 M MgCl_2_, and injected with 20 μl into the opposite direction as SynCom. EA: *E. amylovora*, A: antibacterial, N: network, P: pathway. Disease severity was evaluated using a 0-5 scale based on fruit pictures. Statistical differences among treatments were analyzed with the Kruskal-Wallis test and, if significant, multiple pairwise comparisons were conducted using the Conover-Iman test with BH correction.

In order to observe the overall efficacy of the SynCom control against *E. amylovora* in the Rosaceae family, we conducted a validation of the disease control efficiency of SynCom using rose flowers. Oxytetracycline, a representative chemical pesticide commonly employed in controlling fire blight disease, was used as a positive control to compare and confirm the efficacy of SynCom. In cases where the disease occurred, the seedbed area displayed characteristic darkening and necrosis. Disease severity was assessed by measuring the affected area of the necrotic lesions (Fig. 2A). The group treated solely with the *E. amylovora* exhibited a 100% disease incidence with an approximately 78.33% disease severity index (Fig. 2B). The SynCom treated groups showed varying DSI with EA+A at 40%, EA+A+N at 20%, EA+N+P at 10%, EA+P at 6.67%, EA+A+P at 6.67%, EA+N at 3.33%, EA+A+N+P at 1.67%, EA+oxytetracycline at 1.67%, and control at 0%, in descending order. Conversely, all SynCom strains combined group showed disease severity comparable to the positive control using the oxytetracycline, demonstrating the most effective control. This suggests that the anti-*E. amylovora* effects observed in a controlled environment differ from the results within the plant tissues, indicating varying abilities of different groups to suppress diseases in *planta*.

### SynCom Can Suppress Fire Blight Disease That Occurs in Apple Plants

**Fig. 3:**
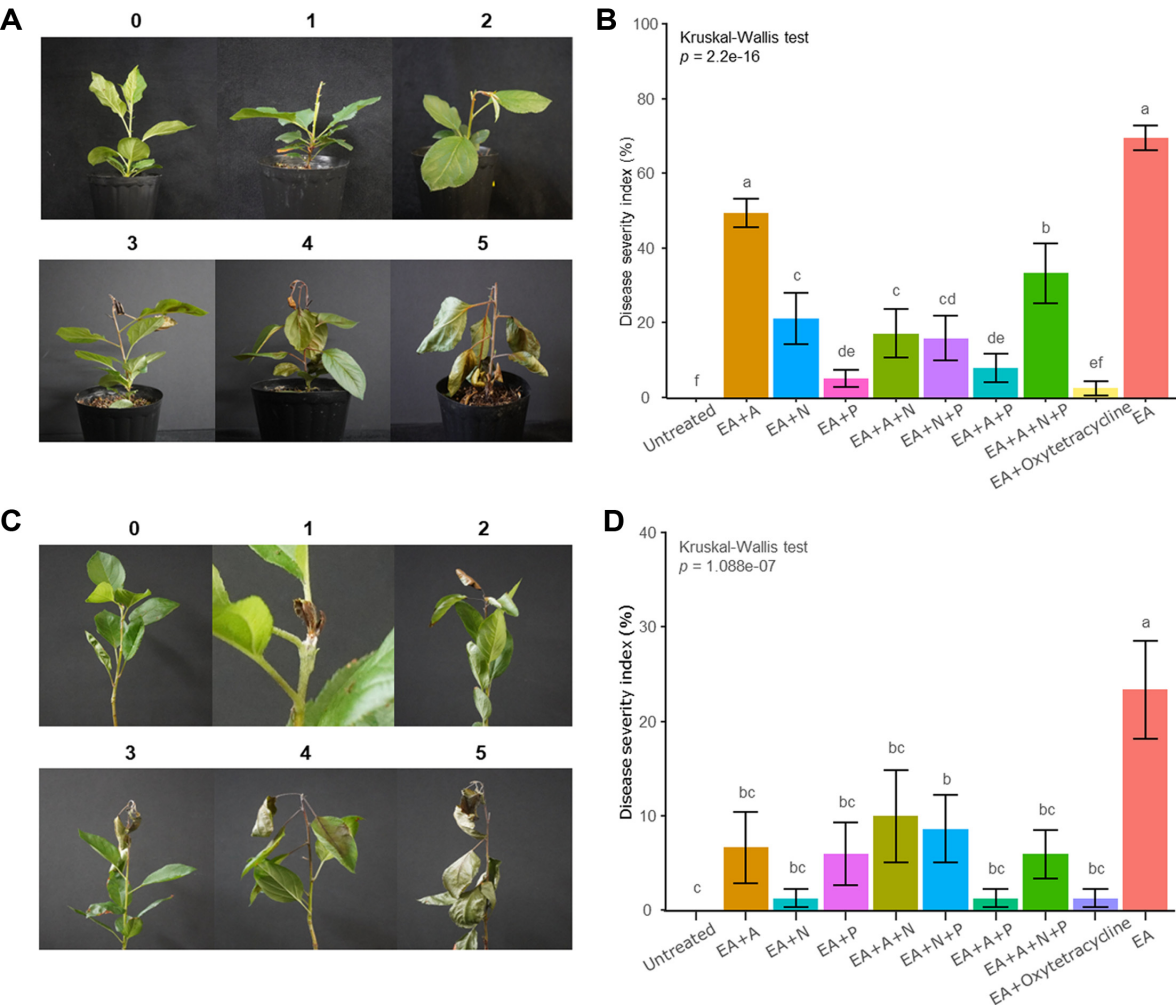
Fire blight disease control by the SynCom. (**A**) Disease severity index in apple plants of the cutting inoculation (*n* = 15). Index 0: No symptoms, Index 1: Partial necrosis of the shoot tip, Index 2: Complete necrosis of the shoot tip, Index 3: Partial necrosis of the terminal leaves, Index 4: Complete necrosis on the petiole of terminal leaves, Index 5: Complete necrosis on the main stem. OD_600_ of 0.2 SynCom strains mixed with 1/10 of 1% CMC, were sprayed onto 15 plant surfaces. After 24 hours, scissors dipped in *E. amylovora* suspension (OD_600_ of 0.2) were used to cut 1st or 2nd leaf stems. (**B**) EA: *E. amylovora*, A: antibacterial, N: network, P: pathway. Disease severity was evaluated 7 days later. Statistical differences were analyzed with the Kruskal-Wallis test and further examined using Conover-Iman tests with BH correction. (**C**) Disease severity index in apple plants of the brushing inoculation (*n* = 30). The assay was repeated by preparing same SynCom mixture and pathogenic suspension. The mixture was applied with a brush to incisions along the plant bark. After the SynCom dried, the same areas were inoculated with the pathogen. (**D**) Represent for assessment of fire blight severity on the brushing inoculation. EA: *E. amylovora*, A: antibacterial, N: network, P: pathway. Statistical analysis was conducted as in (**B**).

The severity of fire blight in apple plants was assessed with an index ranging from 0 to 5 (Fig. 3A and 3C). The severity index of fire blight was calculated as follows: EA 69.4%, EA+A 49.4%, EA+A+N+P 33.4%, EA+N 21.4%, EA+A+N 17.4%, EA+N+P 16%, EA+A+P 8%, EA+P 5.4%, EA+oxytetracycline 2.6%, and control 0% (Fig. 3B). On the plate, the A group exhibited high enzymatic activity and anti-EA activity (Fig. S9). However, identical to the assay in fruits and roses, it did not show the highest effects in apple plants, resulting in a slight decrease in severity. In contrast, the P group demonstrated high efficacy in controlling fire blight in apple plants. When compared to the positive control group treated with oxytetracycline, the P group had no significant difference in severity. The EA+A+N+P group, which showed high efficacy in controlling fire blight in fruits and roses, exhibited the second-lowest efficacy in apple plants, surpassed only by the A group.

The second evaluation in apple plants was conducted using a different inoculation method of SynCom and pathogens. The severity of fire blight was calculated as follows: EA 69.4%, EA+A 49.4%, EA+A+N+P 33.4%, EA+N 21.4%, EA+A+N 17.4%, EA+N+P 16%, EA+A+P 8%, EA+P 5.4%, EA+oxytetracycline 2.6%, and untreated 0% (Fig. 3D). In the second plants assay, contrasting results were observed compared to the first assay. The A group, which exhibited higher severity in the first assay, showed a lower severity, while the P group, which had the lowest severity in the first assay, demonstrated a relatively higher severity. Notably, the A+P group exhibited significantly higher efficacy in SynCom groups in both the first and second trials. The biocontrol assay using plants revealed substantial differences in the outcomes within the same groups for both the first and second challenges. These variable results may depend on SynCom and pathogen inoculation methods.

### Observation of Microbial Composition Changes in Apple Plants by the SynCom Treatment

**Fig. 4:**
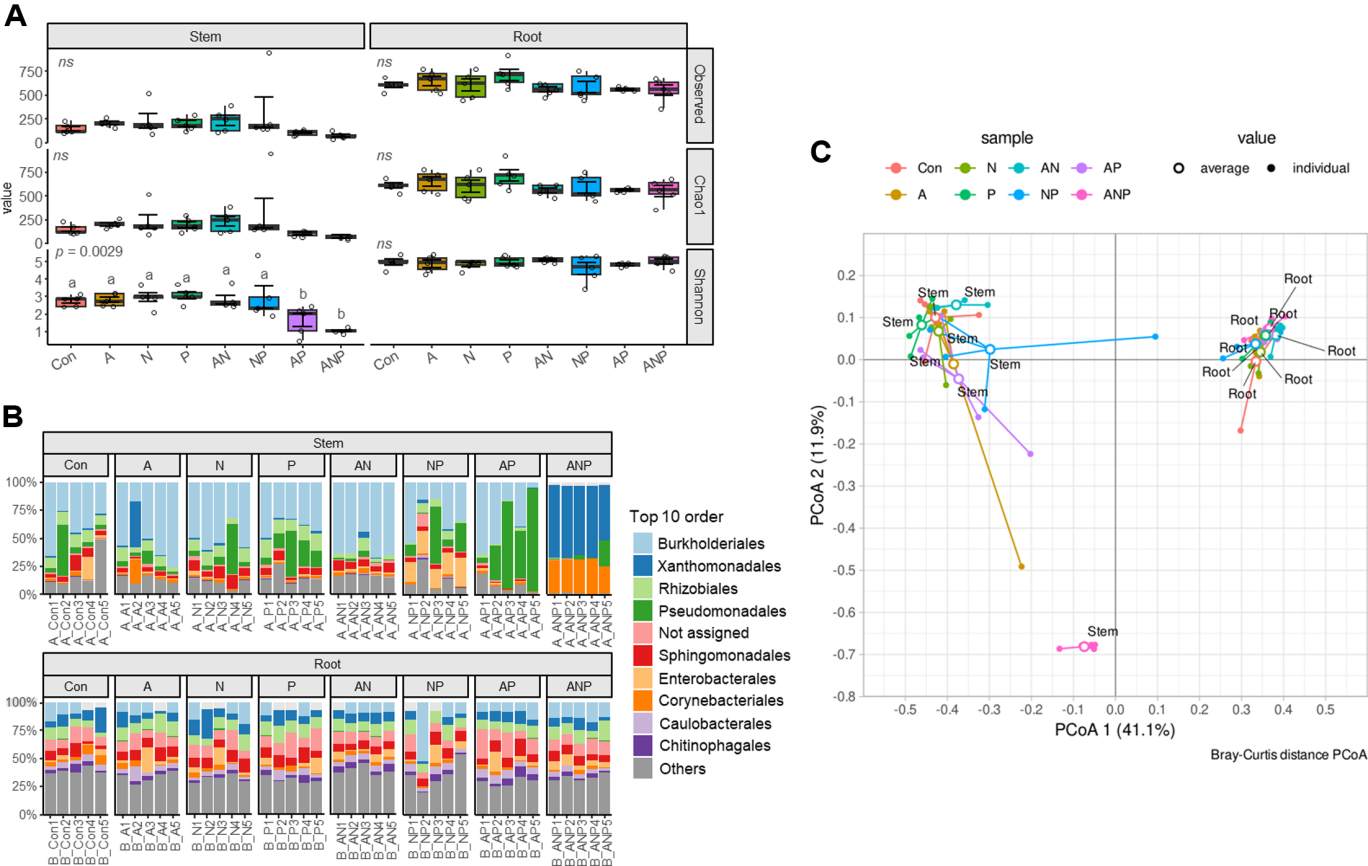
Bacterial diversity and composition across different groups. A: antibacterial, N: network, P: pathway. (**A**) Alpha diversity of bacterial communities for each SynCom group and group combination. Alpha diversity was assessed using the Kruskal-Wallis test, and the letters above the boxes represent the results of the Conover test, indicating differences in alpha diversity values. (**B**) Beta diversity of endophytes in apple plants based on treated groups of SynCom. (**C**) PCoA based on Bray Curtis distance showing the overall composition of bacterial communities based on treated groups of SynCom.

We conducted a comparative analysis of alpha diversity in the endosphere microbial communities of SynCom-treated plants using various indices across different tissues. In the root endosphere, no significant differences in diversity were observed among all SynCom groups, while in stem endosphere, groups with low ASV counts, such as A+P and A+N+P, exhibited lower alpha diversity compared to other SynCom groups in Shannon index (Fig. 4A). Root endophytes showed no substantial differences in the structure and distribution of the top-level microbial communities (Fig. 4B). In contrast, stem endophyte revealed a distinctive pattern of increased density in specific orders. When combining more than two groups in SynCom, the abundance of a specific order increased. When subjected to the N+P group, Enterobacterales exhibited a rising trend in comparison to the control group. Also, the A+P group treatment resulted in an increasing pattern for Pseudomonadales. In the A+N+P group, over 50% of the abundance was attributed to Xanthomonadales, with the remaining abundance dominated by Corynebacteriales. Principal Coordinate Analysis (PCoA) results indicated significant differences among stem endophyte groups compared to the root endophyte, signifying greater microbial compositional variation influenced directly by SynCom in the aboveground parts (Fig. 4C). In contrast, the root endophyte exhibited differences among groups, displaying a more similar pattern compared to the stem endophyte. Particularly, the A+N+P group in stem endosphere demonstrated substantial microbial composition change, indicating its unique presence compared to other groups.

## Discussion

This study aimed to develop reliable control methods against *E. amylovora* by the synthetic microbial community (SynCom), and reduce environmental pollution caused by chemical pesticides, mitigate resistance to bactericides. SynCom, combined multiple strains, serves as a biological control strategy encompassing microbial stability and diversity [35]. SynCom can mimic the functionality and structure of the microbiome present in the environment. In this study, the SynCom constructed consists of 9 strains divided into 3 groups. The groups were selected based on their characteristics: the antibacterial group with high anti-*E. amylovora* activity, the network group with density-dependent correlations in healthy apples, and the pathway group, which metabolic pathways highly expressed in the non-infected apples compared to the *E. amylovora* infected amylovora-infected host plants.

The A (Antibacterial) group selected two strains with antibacterial activity against *E. amylovora*. *Streptomyces recifensis* SN1E1 was isolated from soil [24], while *Paenibacillus polymyxa* AF2927 was isolated from samples in the apple microbiome analysis [21]. The N (network) group consists of strains selected from endophytes present in healthy apple orchards. While some strains may directly inhibit the pathogen through their antibacterial abilities, others with a competitive advantage in terms of space and nutrition within the plant's internal colonization sites can be identified through microbiome analysis [36, 37]. The N group was selected based on SparCC analysis of microbial correlations within tissues collected from healthy orchards in 2020 [22]. SparCC analysis revealed a negative correlation between *E. amylovora* and several genera in density. Three genera, *Labrys* (correlation value: -0.2114), *Novosphingobium* (correlation value: -0.2031), and *Terriglobus* (correlation value: -0.228), exhibiting the high negative correlation were selected, and a total of 5 strains capable of cultivation were obtained from KACC (*Labrys miyagiensis* NBRC101365, *Labrys okinawensis* DSM18385, *Novosphingobium mathurense* SM117, *Novosphingobium endophyticum* EGI60015, *Terriglobus aquaticus* 03SUJ4). On the other hand, the P (pathway) group was formed based on metabolic pathway analysis of non-infected and infected orchards in 2021. We revealed differences in microbial metabolic pathway expression depending on the presence of fire blight in our previous study [20]. PICRUSt2 analysis compared the log2fold change in pathway expression between non-infected and infected orchards [20]. Furthermore, to further validate the correlation between metabolic pathway expression rates and the disease incidence of fire blight, microbial analysis of healthy orchards nationwide in 2022 was conducted [22]. A comparison of pathways between 2021 and 2022 revealed that 11 pathways highly expressed in non-infected orchards in 2021 exhibited similar predicted expression levels in non-infected orchards in 2022. Conversely, it was observed that these metabolic pathways were expressed at lower levels in infected orchards.

The four most highly expressed pathways in healthy orchards were selected, with log2fold change values as follows: toluene degradation III (aerobic) (via p-cresol), 2993.32; catechol degradation I (meta-cleavage pathway), 2454.65; D-galacturonate degradation I, 2896.16; and superpathway of β-D-glucuronosides degradation, 645.45. As a result, two ASVs contributing to these pathways were identified, and *K. papulosa* AF6313, *Pseudomonas lundensis* AB23, which belong to the same genera as the OTUs, were included in the P group. *K. papulosa* AF6313 is a strain that was simultaneously isolated from the endophytic bacterial resource within apple flowers along with *P. polymyxa* AF2927 [22]. On the other hand, *P. lundensis* AB23 was isolated from bee guts in apple orchards and was identified as a closely related species to the OTU selected based on the 16S rRNA V4 region.

The outcomes of our study revealed notable differences between the antibacterial effects of SynCom observed *in planta* and *in vitro*. The A group, which exhibited the highest anti-*E. amylovora* effect *in vitro*, showed high disease incidence and severity on fruits and flowers. Remarkably the A+N+P group, which combined all SynCom strains that had no direct anti-EA effect, showed the highest level of control efficacy on flowers and fruits. This finding indicated that SynCom can be inferred to involve not only direct anti-EA activity but also a variety of disease inhibition mechanisms within plant tissues. Traditionally, efforts have been made to select strains with potential pathogen-inhibiting abilities through *in vitro* conditions using biocontrol assays. However, the effects observed *in vitro* often exhibit variability when translated to field conditions. As an illustration, an assay evaluating the inhibitory capacity of a *Pseudomonas* strain on *Ralstonia solanacearum* in Eucalyptus trees revealed discrepancies between *in vitro* and *in planta* results [38]. It suggested that the lack of consistent efficacy *in planta* could be attributed to lower expression levels of genes responsible for biological control activities [22].

In the case of apple plants, unlike fruits and flowers, the A+N+P group had a relatively low control effect, and there was a large deviation in the results. We speculated that these results might be attributed to resistance mechanisms inherent to the entire plant. Numerous studies provide evidence suggesting the existence of tissue-specific defense mechanisms in plants [39, 40]. Consequently, compared to previous assays using a single tissue, it is inferred that the variability in disease control efficacy observed in plants is primarily due to differences in microbial communities, the inherent health of the plants, and the overall immune responses across various tissues. We also observed differences in disease control efficiency based on inoculation methods in apple plants. Due to the differences in control efficacy depending on whether the SynCom was sprayed on the plants or applied to wounds via brushing, further research is needed to determine the most effective method of SynCom application.

In the analysis of the endophytic microbial communities of plants for stability assessment, it was observed that stem endophyte of the A+N+P group exhibited significantly lower microbial diversity, and a dominance of specific taxa compared to other treatments. This suggests that ensuring microbial diversity within plant tissues might be challenging, posing a potential risk to the stability of microbial diversity when treating all SynCom groups. On the other hand, the pathway group demonstrated a consistently stable control effect across fruits, flowers, and plants. Notably, its efficacy did not diminish when it was used with other groups. It is practically difficult to use too many strains together, and the antagonistic effects among the strains further diminish the feasibility of implementation. Consequently, the pathway group is deemed the most suitable option for preserving control efficacy while maintaining the stability of microbial communities within the ecosystem.

This study demonstrated the effectiveness of SynCom but did not investigate its mechanisms. Recently, there has been a trend toward utilizing genetic engineering strategies for biological control purposes [41, 42]. If the genes of SynCom strains involved in inhibiting or competing against *E. amylovora* are selectively identified and incorporated into a single strain using gene engineering technology as synthetic biology of SynCom. It is conceivable that the same inhibitory effect against the pathogen can be achieved without the need to culture various strains separately. Therefore, elucidating the genetic mechanisms of these SynCom strains could lead to an effective strategy for controlling fire blight disease.

## Supplemental Materials

Supplementary data for this paper are available on-line only at http://jmb.or.kr.



## Figures and Tables

**Table 1:** SynCom selection strains information.

Group	Species	Sources	16S rRNA similarity (%)	Strain	Ref.
Antibacterial	*Streptomyces recifensis*	Soil	98.62	SN1E1	[34]
	*Paenibacillus polymyxa*	Apple flower bud	99.66	AF2927	[21]
Network	*Labrys miyagiensis*	Grassland soil of an experimental farm	99.92	NBRC101365	[43]
	*Labrys okinawensis*	Root-nodule of *Entada phaseoloides*	97.40	DSM18385	
	*Novosphingobium mathurense*	Sea sand soil	100	SM117	[44]
	*Novosphingobium endophyticum*	Roots of *Glycyrrhiza uralensis*	99.92	EGI60015	[45]
	*Terriglobus aquaticus*	Freshwater	99.88	03SUJ4	[46]
Pathway	*Kitasatospora papulosa*	Apple flower	100	AF6313	[21]
	*Pseudomonas lundensis*	Bee gut	99.93	AB23	[21]

**Table 2:** Antibacterial activity of SynCom members against *E. amylovora*.