# Supplementary material for: Design and Evaluation of Synthetic Microbial Communities for Effective Fire Blight Disease Control in Apples
Source: J Microbiol Biotechnol. 2025 Apr 11;35:e2501047. doi: 10.4014/jmb.2501.01047 (PMC12010091; doi:10.4014/jmb.2501.01047)
Supplement: Supplementary file 1 [file jmb-35-e2501047-supple.pdf]

J. Microbiol. Biotechnol. 2025. 35: e2501047
https://doi.org/10.4014/jmb.2501.01047

Supplementary Table and Figures

Design and Evaluation of Synthetic Microbial Communities for Effective Fire Blight Disease Control in Apples

Yejin Lee, Da-Ran Kim, and Youn-Sig Kwak

Table 1. Evaluation of plant growing-promoting effects of SynCom strains.

Group	Strain name	Cellulase production	Protease production	Chitinase production	Siderophore production	Nitrogen fixation	Biofilm production	IAA production	Phosphate solubilization
Antibacterial	SN1E1	++	+	-	-	+	+	-	-
	AF2927	++	++	-	-	-	-	-	+
Network	NBRC101365	-	-	-	-	-	-	++	-
	DSM18385	-	-	-	-	-	++	-	-
	SM117	-	-	-	-	-	-	+	-
	EGI60015	-	-	-	-	-	-	+	-
	03SUJ4	-	-	-	-	-	+	++	-
Pathway	AF6313	+	++	-	-	-	-	-	-
	AB23	-	-	-	-	++	-	-	+

. –: negative, +: Low positive, ++: High positive.

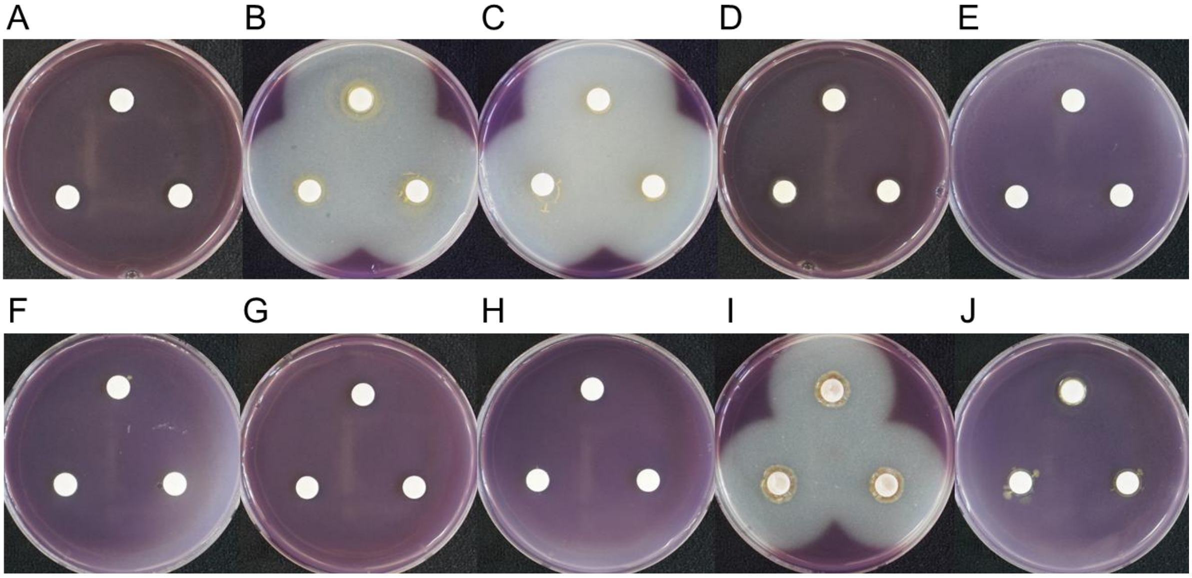

Supplementary Fig. S1. Cellulase production test. A: Control, B: *S. recifensis* SN1E1, C: *P. polymyxa* AF2927, D: *L. miyagiensis* NBRC101365, E: *L. okinawensis* DSM18385, F: *N. mathurense* SM117, G: *N. endophyticum* EGI18385, H: *T. aquaticus* 03SUJ4, I: *K. papulosa* AF6313, J: *P. lundensis* AB23. 20 μL of OD_{600} of 0.5 strains was Inoculated. After incubation at 28 ℃ for 2 days on 1% carboxymethyl cellulose media, Gram's iodine solution (0.67% KI, 0.33% I_2) was poured onto the plate.

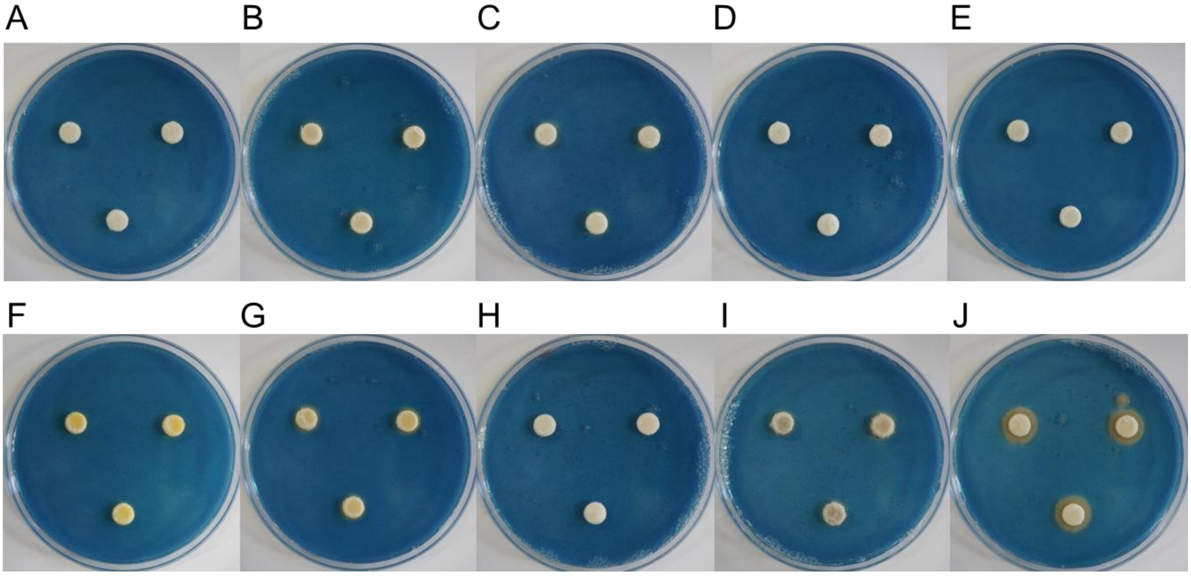

Supplementary Fig. S2. Siderophore production test. A: Control, B: *S. recifensis* SN1E1, C: *P. polymyxa* AF2927, D: *L. miyagiensis* NBRC101365, E: *L. okinawensis* DSM18385, F: *N. mathurense* SM117, G: *N. endophyticum* EGI18385, H: *T. aquaticus* 03SUJ4, I: *K. papulosa* AF6313, J: *P. lundensis* AB23. 20 μL of OD_{600} of 0.5 strains was inoculated on CAS agar plate. After incubation at 28 ℃ for 7 days on CAS agar media, clear zones were measured.

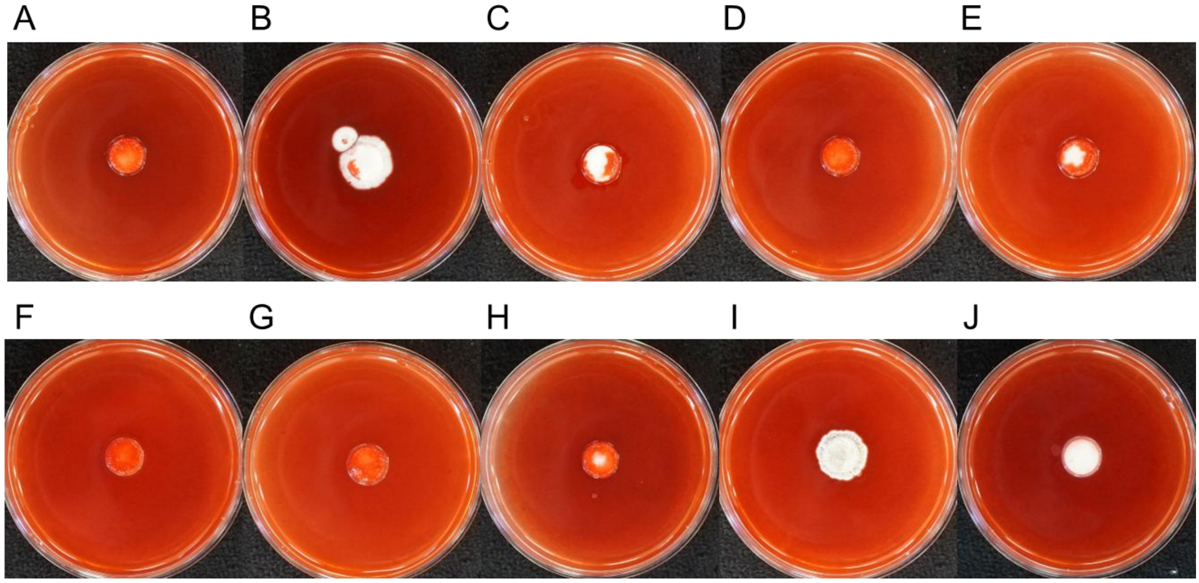

Supplementary Fig. S3. Chitinase production test. A: Control, B: S. recifensis SN1E1, C: P. polymyxa AF2927, D: L. miyagiensis NBRC101365, E: L. okinawensis DSM18385, F: N. mathurense SM117, G: N. endophyticum EGI18385, H: T. aquaticus 03SUJ4, I: K. papulosa AF6313, J: P. lundensis AB23. 20 μL of strains with an OD600 of 0.3 were inoculated on chitinolytic reaction media. After incubation at 28 ℃ for 3 days, 1 mL of 0.1% Congo Red was dispensed per plate and incubated for 15-30 min. Then, the plate was washed twice by dispensing 2 mL of 1M NaCl and the clear zone was measured.

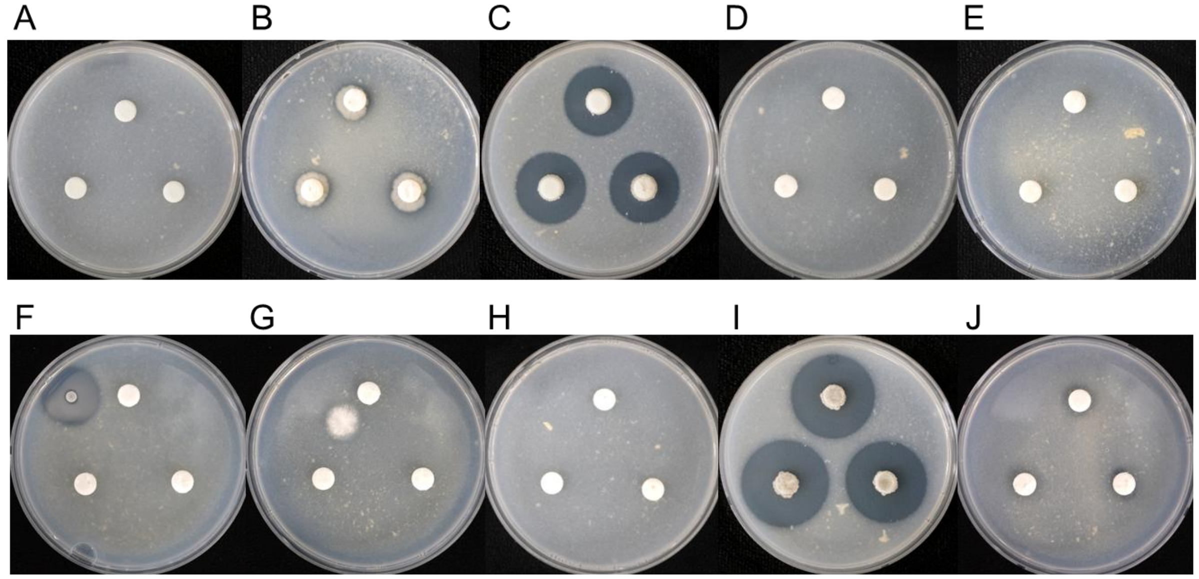

Supplementary Fig. S4. Protease production test. A: Control, B: *S. recifensis* SN1E1, C: *P. polymyxa* AF2927, D: *L. miyagiensis* NBRC101365, E: *L. okinawensis* DSM18385, F: *N. mathurense* SM117, G: *N. endophyticum* EGI18385, H: *T. aquaticus* 03SUJ4, I: *K. papulosa* AF6313, J: *P. lundensis* AB23. 20 μL of OD_{600} of 0.5 strains was Inoculated. After incubation at 28 ℃ for 3 days on skim milk agar media, clear zones were measured.

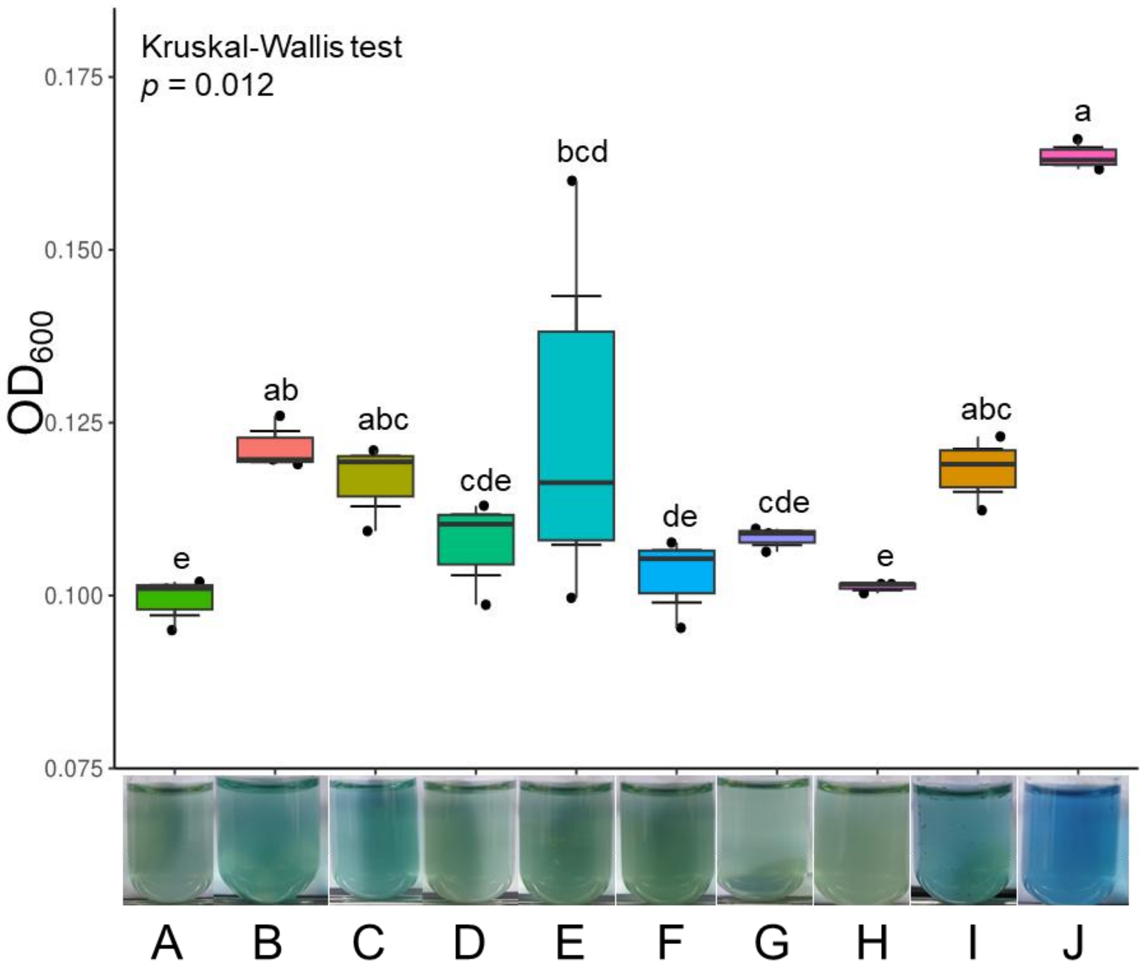

Supplementary Fig. S5. Nitrogen fixation test. A: Control, B: *S. recifensis* SN1E1, C: *P. polymyxa* AF2927, D: *L. miyagiensis* NBRC101365, E: *L. okinawensis* DSM18385, F: *N. mathurense* SM117, G: *N. endophyticum* EGI18385, H: *T. aquaticus* 03SUJ4, I: *K. papulosa* AF6313, J: *P. lundensis* AB23. 5 mL of NFB media was inoculated and cultured at 28 ℃ for 3 days.

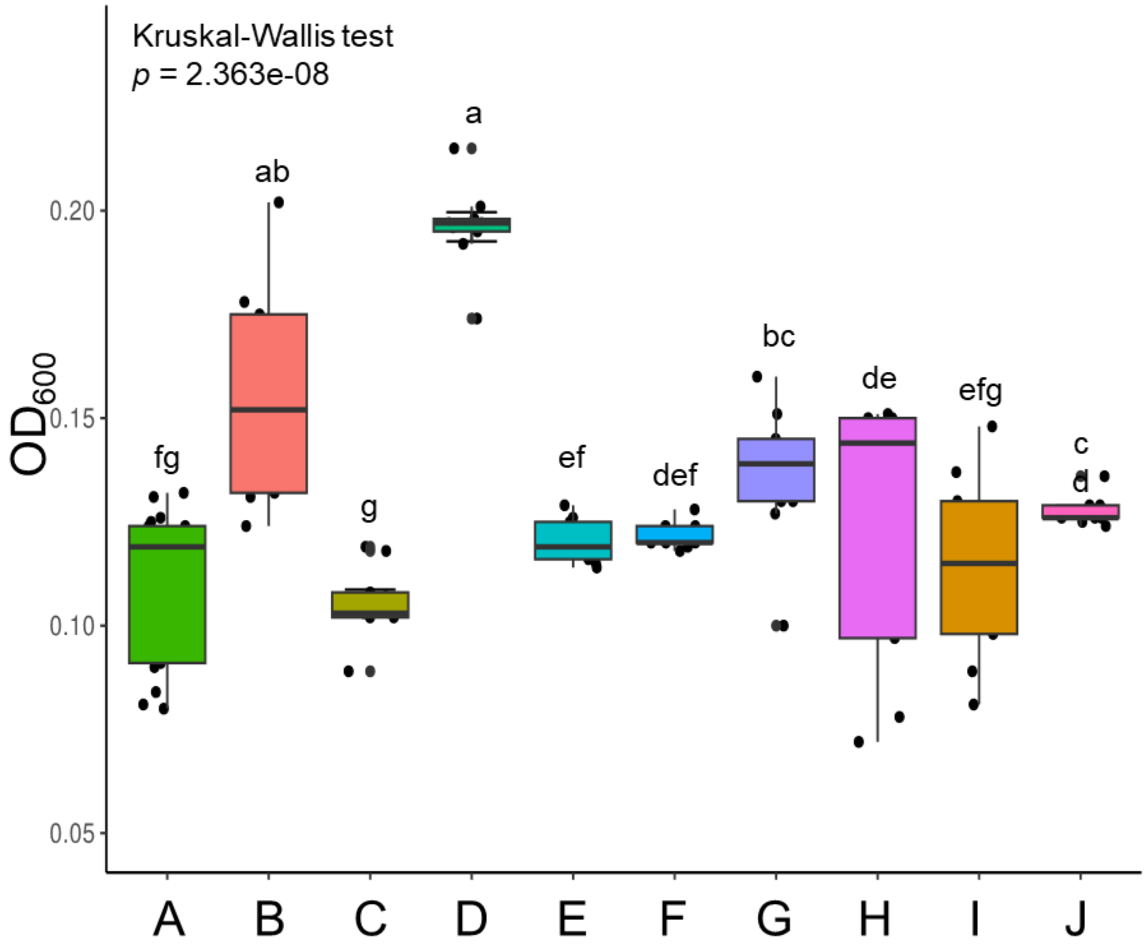

Supplementary Fig. S6. Biofilm production test. A: untreated, B: *S. recifensis* SN1E1, C: *P. polymyxa* AF2927, D: *L. miyagiensis* NBRC101365, E: *L. okinawensis* DSM18385, F: *N. mathurense* SM117, G: *N. endophyticum* EGI18385, H: *T. aquaticus* 03SUJ4, I: *K. papulosa* AF6313, J: *P. lundensis* AB23. M63 media was inoculated and cultured at 28 ℃ for 24 h. Wells were washed and stained with 0.1% crystal violet for 10 min. Then crystal violet dissolved in 95% ethanol.

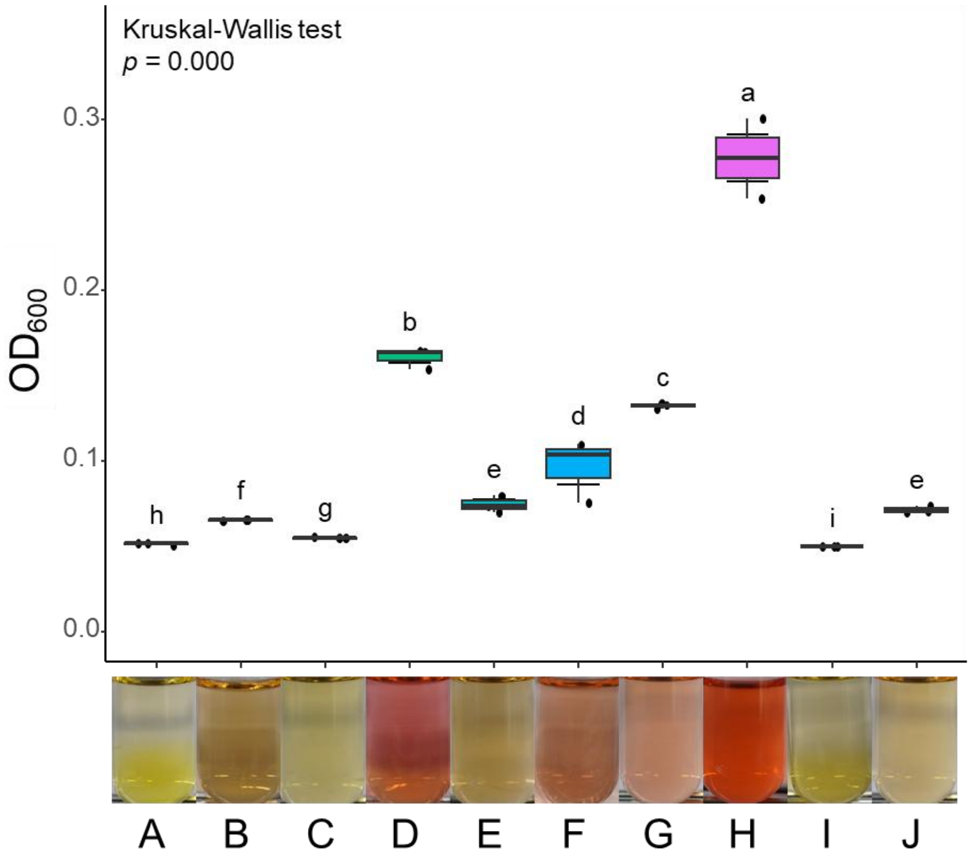

Supplementary Fig. S7. IAA production test. A: Control, B: *S. recifensis* SN1E1, C: *P. polymyxa* AF2927, D: *L. miyagiensis* NBRC101365, E: *L. okinawensis* DSM18385, F: *N. mathurense* SM117, G: *N. endophyticum* EGI18385, H: *T. aquaticus* 03SUJ4, I: *K. papulosa* AF6313, J: *P. lundensis* AB23. Strains were cultured in R2A+0.5% mannitol media at 30 °C for 3 days.

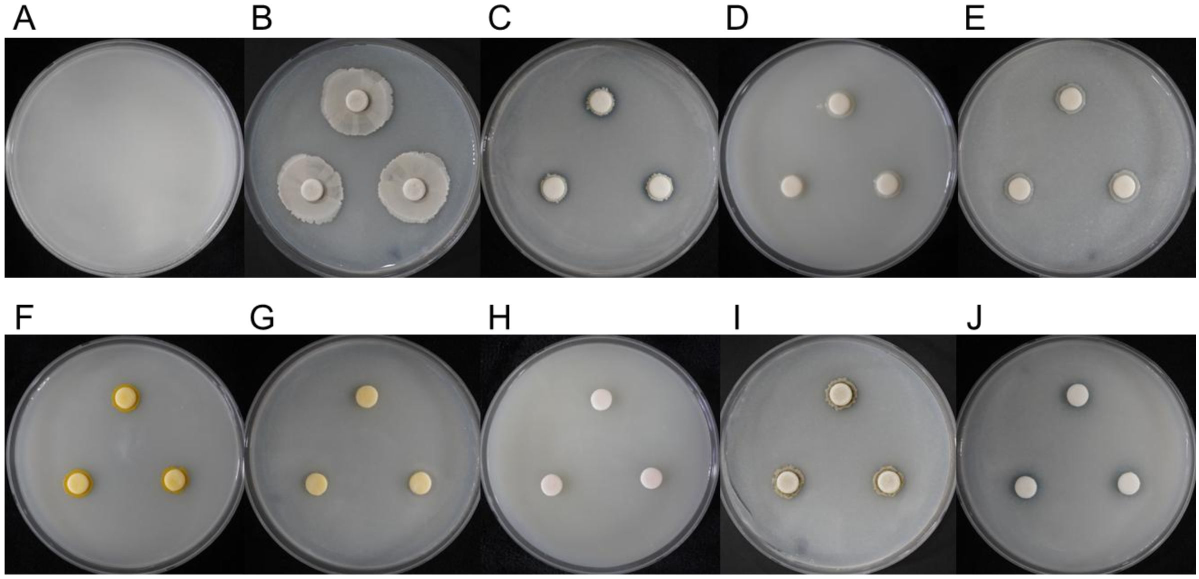

Supplementary Fig. S8. Phosphate solubilization test. A: Control, B: *S. recifensis* SN1E1, C: *P. polymyxa* AF2927, D: *L. miyagiensis* NBRC101365, E: *L. okinawensis* DSM18385, F: *N. mathurense* SM117, G: *N. endophyticum* EGI18385, H: *T. aquaticus* 03SUJ4, I: *K. papulosa* AF6313, J: *P. lundensis* AB23. 20 μL of OD_{600} of 0.5 strains were inoculated. After incubation at 28 ℃ for 2 weeks.

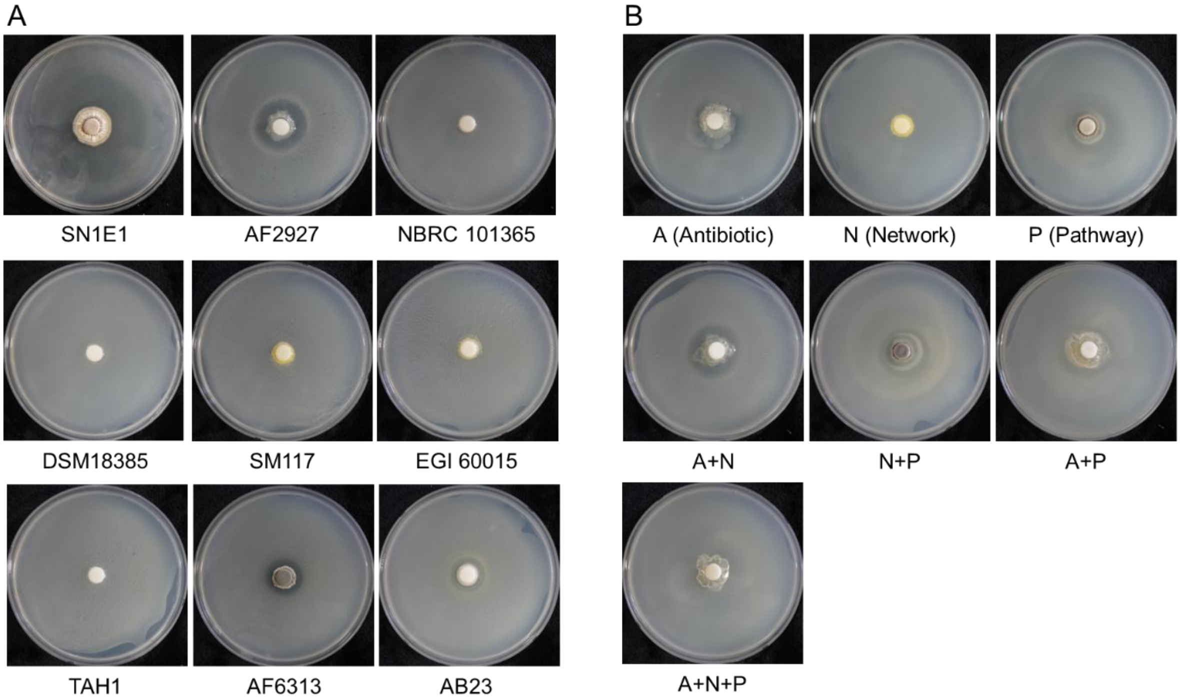

Supplementary Fig. S9. Antibacterial activities against *E. amylovora*. In the antibacterial assay, SynCom strains were cultured in R2A media+ 0.5% mannitol at 28 ℃ for 2 days until reaching an OD_{600} of 0.3. *E. amylovora* was adjusted to an OD_{600} of 0.3 and mixed in a 1:1 ratio with 0.2% agarose, then overlaid onto the culture. The clear zones were measured after 2 days of overlay with *E. amylovora*.
